# Supplementary material for: Impact of Static Distortion Waves on Superlubricity
Source: ACS Omega. 2023 Oct 31;8(45):42457–66. doi: 10.1021/acsomega.3c05044 (PMC10652266; doi:10.1021/acsomega.3c05044)
Supplement: Supplementary file 1 — ao3c05044_si_001.pdf [file ao3c05044_si_001.pdf]

The Impact of Static Distortion Waves on Superlubricity

Supporting information

Lukas Hörmann,* Johannes J. Cartus, and Oliver T. Hofmann

Institute of Solid State Physics, Graz University of Technology, Graz

E-mail: hoermann@tugraz.at

Machine-learned interatomic potentials

As explained in the main manuscript, we use a machine-learned interatomic potential (MLIP) based on Gaussian process regression and SOAP descriptors.[1] A flowchart of our approach is presented in Figure S1 To train the MLIP for a given system we use a two-step procedure: (A) In the first step we train a first MLIP ($\mathrm{MLIP}^1_{\text{mol-sub}}$) for the molecule-substrate interactions. In the case of physisorbed TCP on Pt(111), we train an additional MLIP ($\mathrm{MLIP}^1_{\text{mol-mol-gas}}$) on the molecule-molecule interactions of a free-standing layer of molecules in vacuum. The $\mathrm{MLIP}^1_{\text{mol-sub}}$ (together with the $\mathrm{MLIP}^1_{\text{mol-mol-gas}}$) provides approximate predictions of the formation energies $E^{\text{approx}}_{\text{form}}$ of densely packed surface structures. (B) In the second step we determine the residual between the approximate predictions and formation energies that were calculated with DFT. We then train another MLIP ($\mathrm{MLIP}^2_{\text{conf}}$) on these residuals. The combination of MLIP^1_{*} and $\mathrm{MLIP}^2_{\text{conf}}$ allows us to predict formation energies E_{form} with high accuracy while at the same time reducing the amount of training data.

To train the $\mathrm{MLIP}^1_{\text{mol-sub}}$ we provide calculations of the adsorption energy of a single, isolated molecule (approximated in periodic boundary-conditions DFT by a large unit cell containing one molecule) that assumes different positions and orientations (around the z-axis) on the surface. For the $\mathrm{MLIP}^1_{\text{mol-mol-gas}}$ we provided calculations of densely packed structures of free-standing molecules in vacuum. To train the $\mathrm{MLIP}^2_{\text{conf}}$ we provide calculations of the formation energy of densely packed surface structures. To select the number of training data points, we define a target accuracy of 40 meV, which corresponds to the often-used chemical accuracy for 1 kcal mol^{-1}. In the following subsections, we show the accuracy of our MLIPs.

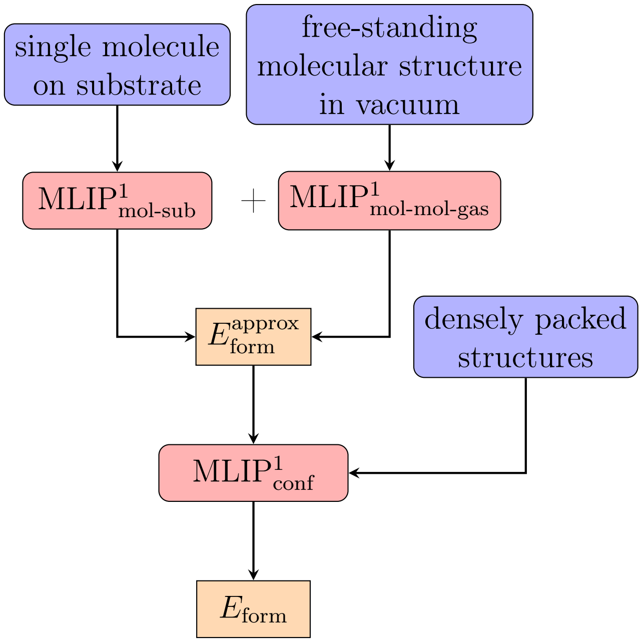

Figure S1: Flowchart of the different MLIPs used to predict the formation energy E_{form}

Naphthalene on Cu(111)

The learning curve of the $\mathrm{MLIP}^1_{\text{mol-sub}}$ is shown in Figure S2. The $\mathrm{MLIP}^1_{\text{mol-sub}}$ reaches a leave-one-out-cross-validation-root-mean-square-error (LOOCV) of 13 meV which is lower than the target accuracy of 40 meV, which corresponds to the often used chemical accuracy for 1 kcal mol^{-1}. For this, we provided 48 training data points.

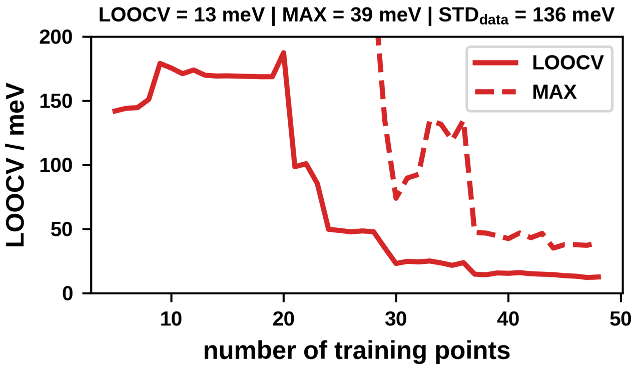

Figure S2: Learning curve of the MLIP for a single molecule of naphthalene on Cu(111)

We do not include an $\mathrm{MLIP}^1_{\text{mol-mol-gas}}$ since our previous work[2] has shown that the character of the molecule-molecule interactions changes due to the interaction with the substrate. More specifically, molecules in a

free-standing monolayer exhibit attractive vdW-interactions. On the Cu(111) substrate naphthalene is slightly charged leading to electrostatic repulsion of the molecules.

The learning curve of the $\mathrm{MLIP}^2_{\mathrm{conf}}$ is shown in Figure S3. It reaches a very low LOOCV of 4 meV which is lower than the target accuracy of 40 meV. For this, we provided 259 training data points. The learning curve in Figure S3 shows only a small improvement when adding more data. We attribute this to the weak and uniform (and thus easy to learn) molecule-molecule interactions of naphthalene on Cu(111). Therefore, most of the relevant information is already captured by the prior for the molecule-substrate interactions. This becomes evident when looking at the standard deviation of the training data set $\mathrm{STD}_{\mathrm{data}}$ in Figure S3, which amounts to only 16 meV.

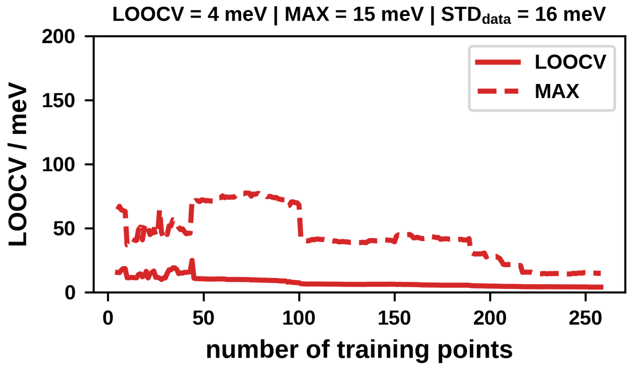

Figure S3: Learning curve of the MLIP for densely packed structures of naphthalene on Cu(111)

Physisorbed TCP on Pt(111)

The learning curve of the $\mathrm{MLIP}^1_{\mathrm{mol\text{-}sub}}$ is shown in Figure S4. This MLIP reaches an LOOCV of 15 meV which is lower than the target accuracy of 40 meV. For this, we provided 50 training data points.

Figure S5 shows the learning curve of the $\mathrm{MLIP}^1_{\mathrm{mol\text{-}mol}}$. We reach an LOOCV of 8 meV which is lower than the target accuracy of 40 meV. The number of training data points is in this case 999. The inclusion of an $\mathrm{MLIP}^1_{\mathrm{mol\text{-}mol}}$ yielded significant improvements in MLIP accuracy in previous work.[3] This is

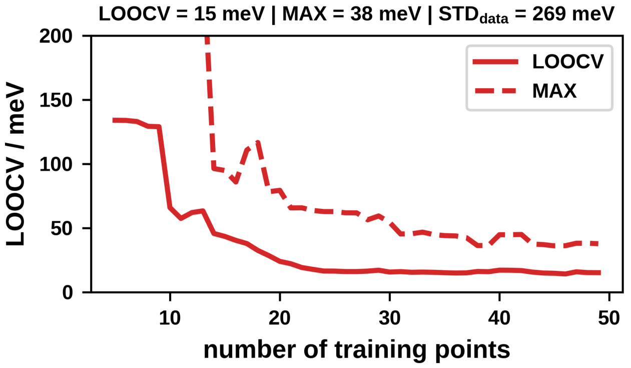

Figure S4: Learning curve of the MLIP for a single molecule of physisorbed TCP on Pt(111)

due to the strong molecule-molecule interactions whose character remains relatively unchanged due to interactions with the substrate.

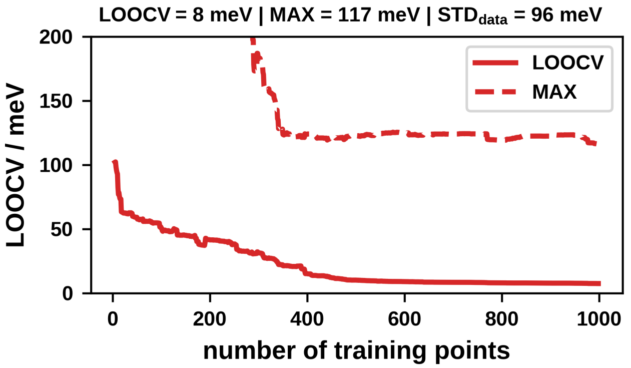

Figure S5: Learning curve of the MLIP for a free-standing monolayer of TCP molecules in vacuum

The learning curve of the $\mathrm{MLIP}^2_{\mathrm{conf}}$ is shown in Figure S6. It reaches a very low LOOCV of 19 meV and therefore reaches the target accuracy of 40 meV. To reach this accuracy, we trained the MLIP on 50 densely packed structures. Similar to naphthalene on Cu(111), the learning curve in Figure S6 shows minor improvement when adding more data. This is likely due to most of the relevant information being already included in $\mathrm{MLIP}^1_{\mathrm{mol\text{-}sub}}$ and $\mathrm{MLIP}^1_{\mathrm{mol\text{-}mol}}$. This assumption is rooted in the fact that physisorbed TCP is physisorbed and that the corrugation of its molecule-substrate interaction is very small (see Figure 1 in the main manuscript). Moreover, the standard deviation of the training data ($\mathrm{STD}_{\mathrm{data}}$=20 meV) is small. We note that

accurate predictions could be made with only $\text{MLIP}^1_{\text{mol-sub}}$ and $\text{MLIP}^1_{\text{mol-mol}}$. However, we use $\text{MLIP}^2_{\text{conf}}$ (despite the very small improvement) to retain a consistent workflow for all systems.

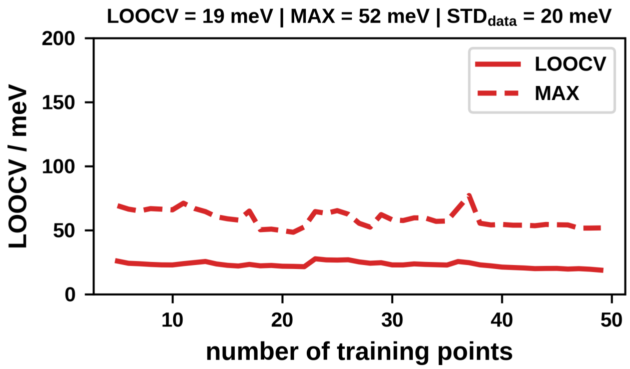

Figure S6: Learning curve of the MLIP for densely packed structures of physisorbed TCP on Pt(111)

Chemisorbed TCP on Pt(111)

The learning curve of the $\text{MLIP}^1_{\text{mol-sub}}$ is shown in Figure S7. It reaches a LOOCV of 35 meV which is lower than the target accuracy of 40 meV. For this, we provided 80 training data points. Chemisorbed TCP exhibits the most corrugated PES of the molecule-substrate interactions as evidenced by the large standard deviations of the data of STD_{data}=731 meV.

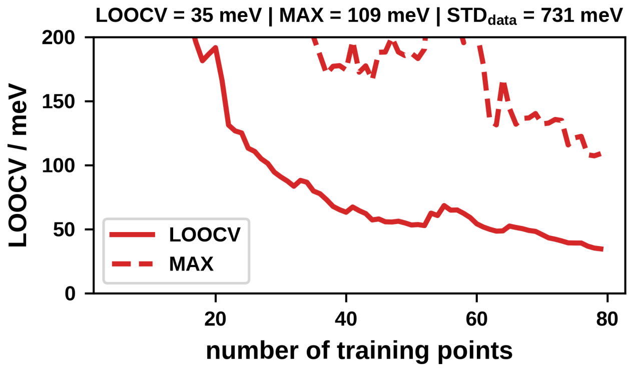

Figure S7: Learning curve of the MLIP for a single molecule of chemisorbed TCP on Pt(111)

We do not include an $\text{MLIP}^1_{\text{mol-mol}}$ since our previous work[3] has shown that the character of the molecule-molecule interactions changes due to the strong interaction with the substrate. This is due to strong deformations of the chemisorbed TCP molecules.

We show the learning curve of the $\text{MLIP}^2_{\text{conf}}$ in Figure S8. It reaches a very low LOOCV of 38 meV which is lower than the target accuracy of 40 meV. For this, we provided 100 training data points of densely packed structures.

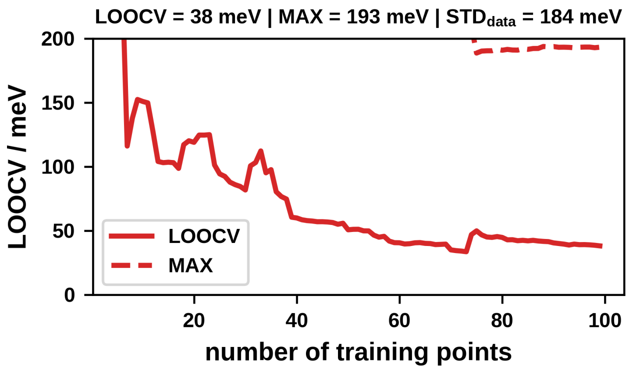

Figure S8: Learning curve of the MLIP for densely packed structures of chemisorbed TCP on Pt(111)

Comparison of the MLIP to previous machine-learning models

To ensure that our MLIP produces predictions of the same quality compared to the previous machine-learning models we calculate the prediction errors for the formation energy of adsorbate structures between the old models and the MLIP. For Naphthalene on Cu(111) we use a subset of 9868 configurations and compare the predictions made with SAMPLE[2] to those made with the MLIP. For chemisorbed and physisorbed TCP on Pt(111) we use subsets containing 10 096 and 11 404 structures respectively and compare the predictions made with a Gaussian process regression (GPR) model using descriptors based on radial distance function (RDF)[3] to predictions with the MLIP. The results are shown in Figures S9 to S11. For all systems we determine an root-mean-square-error (RMSE): For naphthalene on Cu(111) the formation energy for the MLIP and the SAMPLE method differ on average by 0.1 meV per molecule. For physisorbed TCP on Pt(111) the formation energy for the MLIP

and the GPR+RDF method differ on average by 0.3 meV per molecule, while for difference for chemisorbed TCP is 0.6 meV per molecule. These differences in formation energy predictions are small compared to our target uncertainty of 40 meV per molecule. We note in passing that the largest difference in formation energy prediction amounts to approximately 100 meV per molecule which is in order of magnitude of the maximal errors of the MLIP.

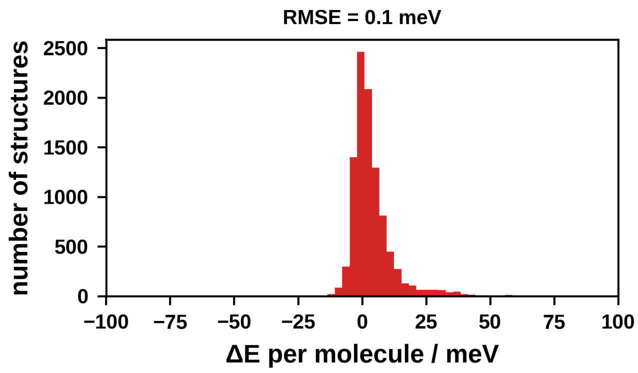

Figure S9

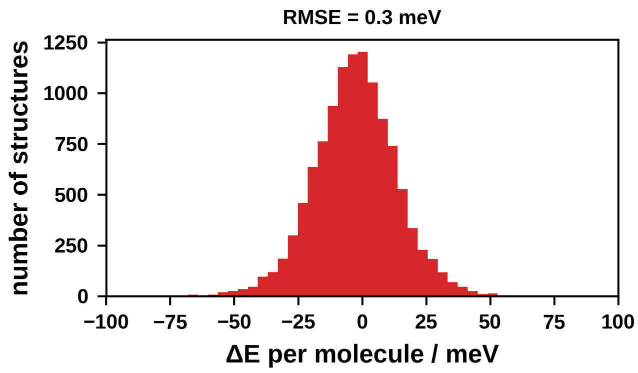

Figure S10

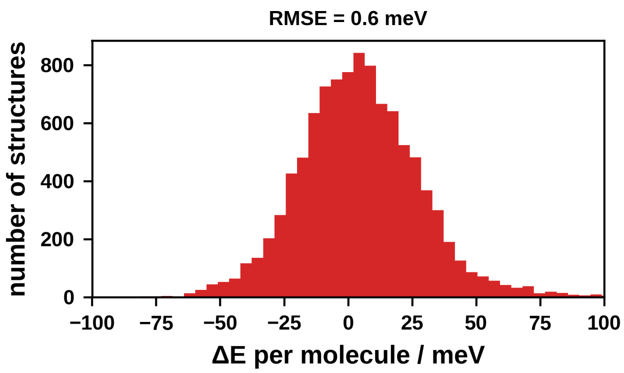

Figure S11

Molecule-molecule interactions

The strength of the molecule-molecule interactions has a crucial impact on the type of commensurability of an adlayer structure.[4,5] The molecule-molecule interactions depend on a variety of factors, which include the chemical composition and structure of the molecules, possible geometric distortions of the molecules, the amount of orbital hybridization with the substrate or the charge transferred between molecules and substrate. The adsorption height of the molecules impacts the interaction between the molecule and the substrate which in turn governs geometric distortions, hybridization and charge transfer. To gauge the impact of the adsorption height on the molecule-molecule interactions, we have calculated molecule-molecule interaction maps at heights that are 0.1 Å lower as well as 0.1 Å higher than the average height. For all three systems, the molecule-molecule interaction at lower-than-average, average and higher-than-average adsorption heights show only marginal differences. The results are shown in Figure S12. In the case of the physisorbed molecules (see Figure S12a,c,d,f,g,i) the change in adsorption height has almost no impact on the molecule-molecule interaction. Conversely, a small impact can be seen in the case of chemisorbed TCP (see Figure S12b,e,h).

Convergence of the unit cell size of pseudo-incommensurate structures

Figures S13 and S14 show the dependence of the static friction coefficient on the number of molecules in the unit cell for naphthalene on Cu(111) and TCP on Pt(111) respectively. We find a decrease in the static friction coefficient

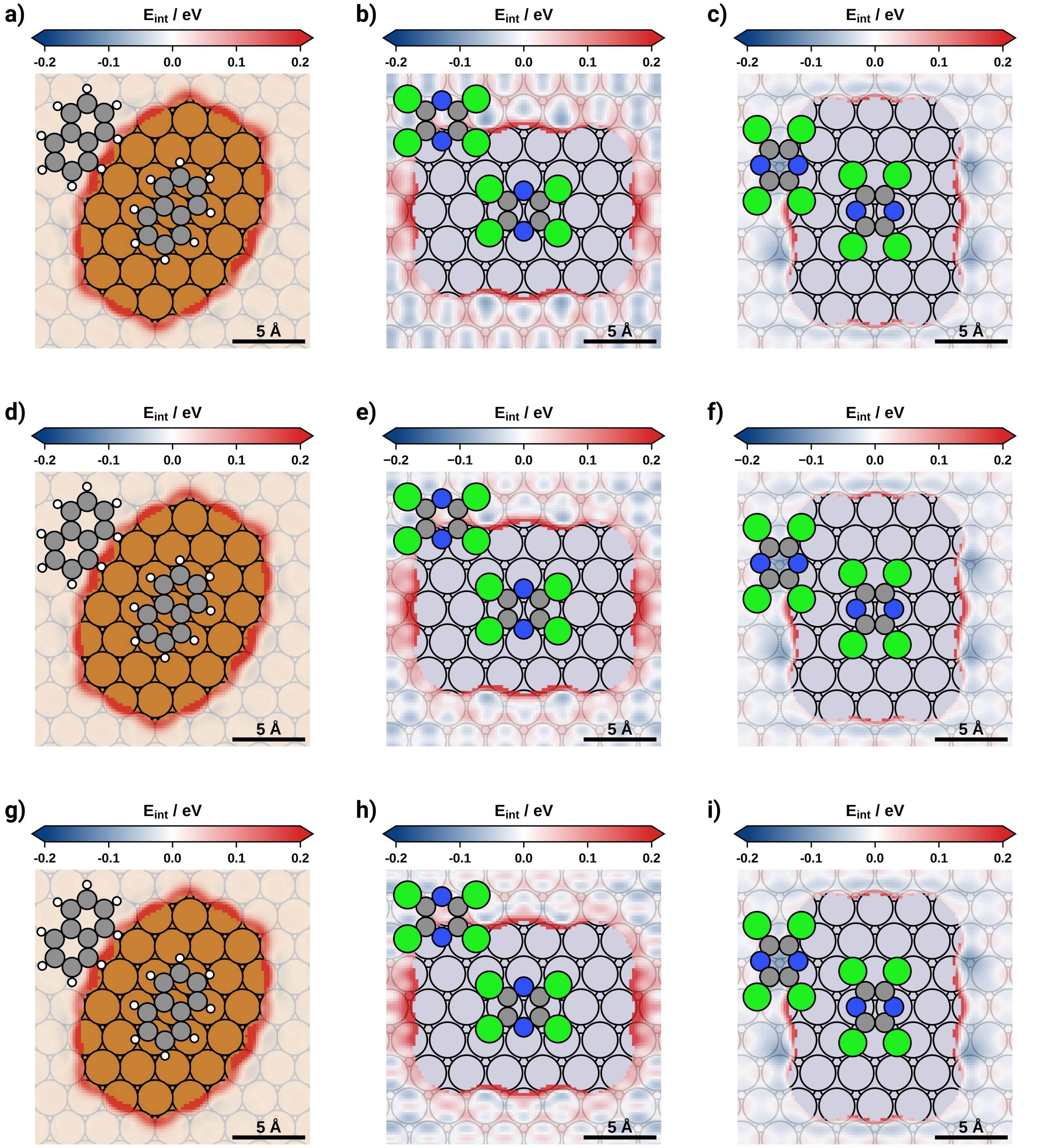

Figure S12: Molecule-molecule interaction if the molecules are at a height that is a,b,c) 0.1 Å lower than the average height $\langle h \rangle$, d,e,f) at average height $\langle h \rangle$ and g,h,i) at a height that is 0.1 Å higher than $\langle h \rangle$

that is slower than exponential. Therefore, even an increase of the unit cell size by an order of magnitude, which could no longer be calculated with our methodology, would not lead to a significant decrease in the static friction coefficient. We note in passing that a truly incommensurate unit cell, containing an infinite number of molecules at different adsorption sites, would yield a static friction coefficient of 0. However, we use the pseudo-incommensurate unit cells as a reference point in our study and to show trends. Therefore, the finding of our study is not impacted by the finite size of the pseudo-incommensurate unit cells.

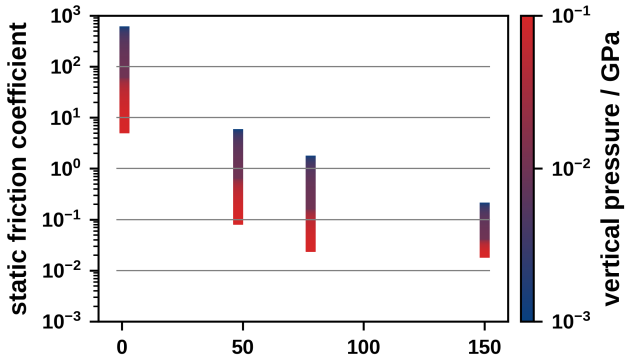

Figure S13: Convergence of the static friction coefficient with respect to the size of the pseudo-incommensurate unit cell of naphthalene on Cu(111)

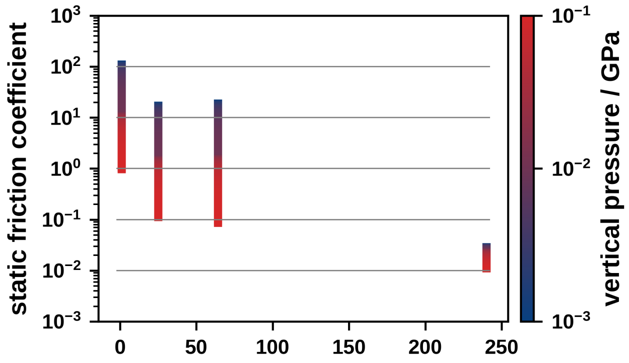

Figure S14: Convergence of the static friction coefficient with respect to the size of the pseudo-incommensurate unit cell of TCP on Pt(111)

Figure S15 and S16 show the pseudo-incommensurate structures of naphthalene on Cu(111) and TCP on Pt(111) respectively.

References

(1) Bartók, A. P.; Kondor, R.; Csányi, G. On representing chemical environments. *Physical Review B* **2013**, *87*, 184115.

(2) Hörmann, L.; Jeindl, A.; Egger, A. T.; Scherbela, M.; Hofmann, O. T. SAMPLE: Surface structure search enabled by coarse graining and statistical learning. *Computer Physics Communications* **2019**, *244*, 143–155.

(3) Hörmann, L.; Jeindl, A.; Hofmann, O. T. From a bistable adsorbate to a switchable interface: tetrachloropyrazine on Pt (111). *Nanoscale* **2022**, *14*, 5154–5162.

(4) Hooks, D. E.; Fritz, T.; Ward, M. D. Epitaxy and Molecular Organization on Solid Substrates. *Adv. Mater.* **2001**, *13*, 227–241.

(5) Mannsfeld, S.; Fritz, T. Understanding Organic–Inorganic Heteroepitaxial Growth of Molecules on Crystalline Substrates: Experiment and Theory. *Phys. Rev. B* **2005**, *71*, 235405.

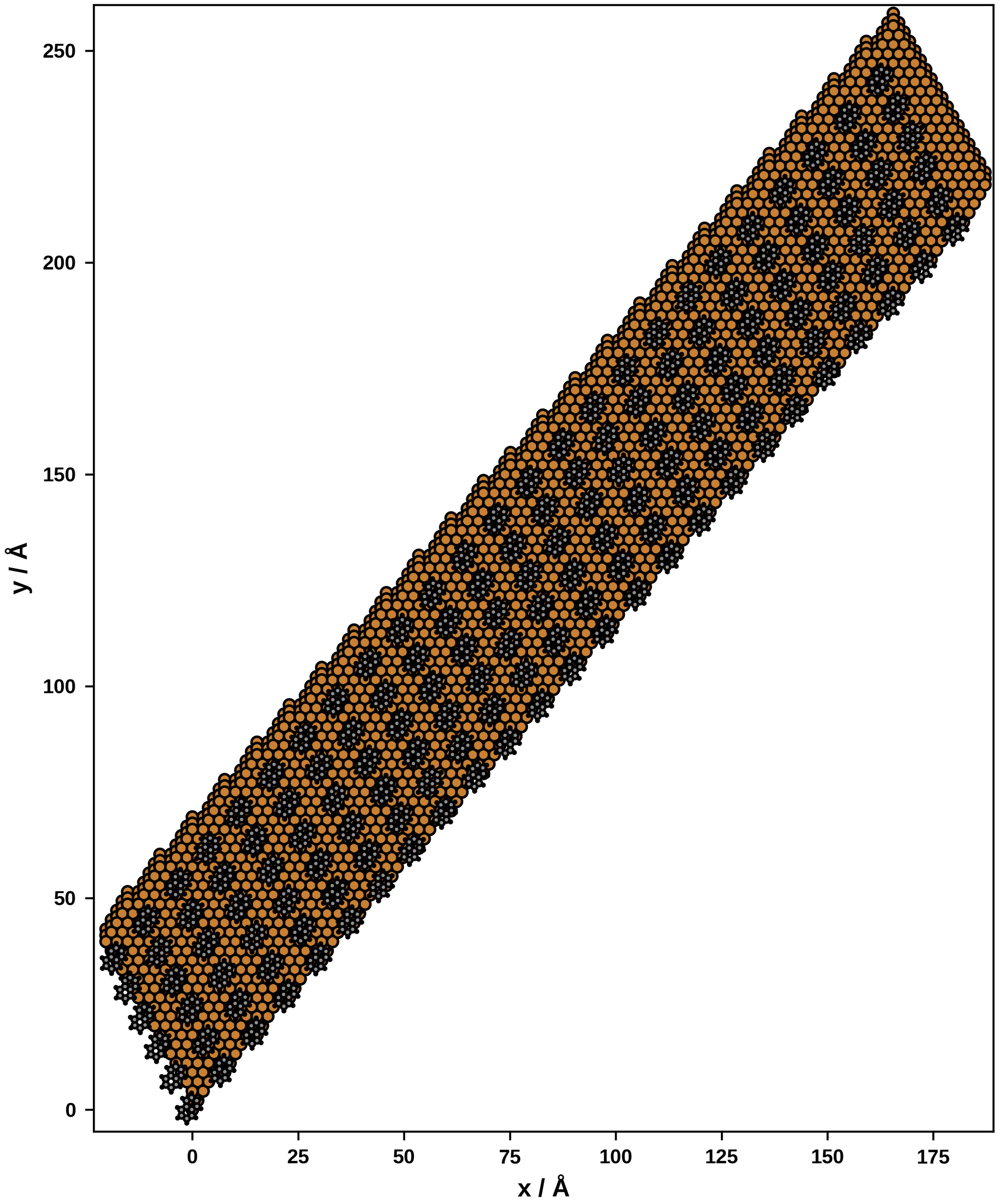

Figure S15

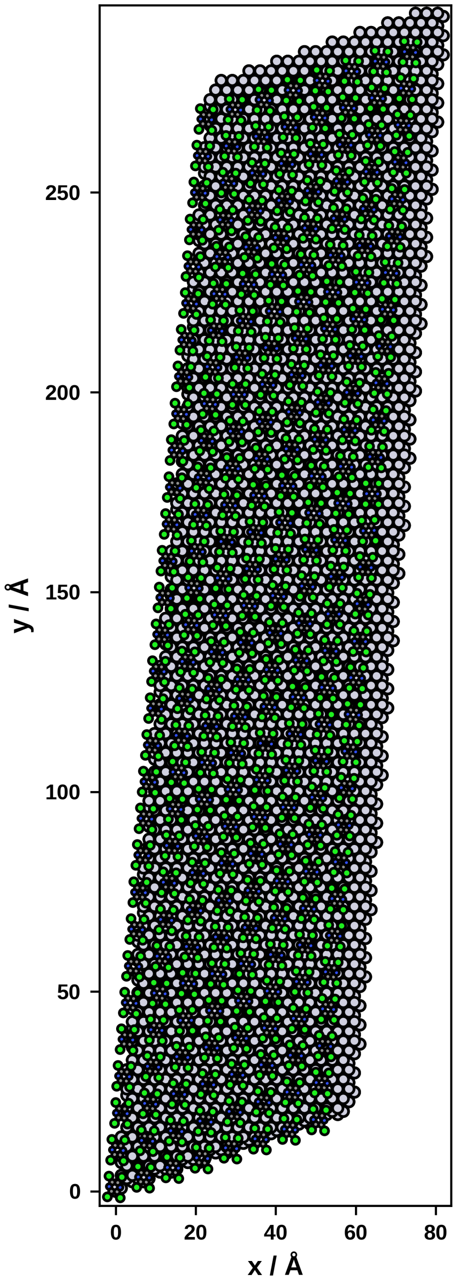

Figure S16
